# Parkinsonian Rigidity Shows Variable Properties Depending on the Elbow Joint Angle

**DOI:** 10.1155/2013/258374

**Published:** 2013-01-27

**Authors:** Takuyuki Endo, Toshimitsu Hamasaki, Ryuhei Okuno, Masaru Yokoe, Harutoshi Fujimura, Kenzo Akazawa, Saburo Sakoda

**Affiliations:** ^1^Department of Neurology, Toneyama National Hospital, 5-1-1 Toneyama, Osaka, Toyonaka 560-8552, Japan; ^2^Department of Biomedical Statistics, Osaka University Graduate School of Medicine, 2-2 Yamadaoka, Osaka, Suita 565-0879, Japan; ^3^Department of Electrical and Electronic Engineering, Setsunan University, 17-8 Ikedanaka-machi, Osaka, Neyagawa 572-8508, Japan; ^4^Department of Neurology, Osaka University Graduate School of Medicine, 2-2 Yamadaoka, Osaka, Suita 565-0879, Japan; ^5^Department of Biomedical Engineering, Osaka Institute of Technology, 5-16-1 Omiya, Osaka, Asahi-ku 535-8585, Japan

## Abstract

Parkinsonian rigidity has been thought to be constant through a full range of joint angle. The aim of this study was to perform a detailed investigation of joint angle dependency of rigidity. We first measured muscle tone at the elbow joint in 20 healthy subjects and demonstrated that an angle of approximately 60° of flexion marks the division of two different angle-torque characteristics. Then, we measured muscle tone at the elbow joint in 24 Parkinson's Disease (PD) patients and calculated elastic coefficients in flexion and extension in the ranges of 10°–60° (distal) and 60°–110° (proximal). Rigidity as represented by the elastic coefficient in the distal phase of elbow joint extension was best correlated with the UPDRS rigidity score (*r* = 0.77). A significant difference between the UPDRS rigidity score 0 group and 1 group was observed in the elastic coefficient in the distal phase of extension (*P* < 0.0001), whereas no significant difference was observed in the proximal phase of extension and in each phase of flexion. Parkinsonian rigidity shows variable properties depending on the elbow joint angle, and it is clearly detected at the distal phase of elbow extension.

## 1. Introduction

Parkinsonian rigidity has been thought to be constant through a full range of joint angle [1]. However, such definition is based on the subjective observation, and it is unclear whether rigidity varies with joint angles or not. We have successfully analyzed the components of rigidity perceived by physicians in routine clinical practice in a systematic way [2]. As a result, we found that rigidity consists of elastic coefficients (elasticity) and difference of bias (difference in torque measurements for extension and flexion) and that higher values of either or both of these components are associated with greater amounts of rigidity. The elastic coefficients in flexion and extension of the elbow joint were estimated by extracting the 10°–110° portion of the degree-torque characteristics curve and using the slope of the regression line relative to the entire data. Further analysis of the data of healthy subjects demonstrated that the 60° elbow joint angle marks the division of the two properties of the elastic coefficients. We considered the elbow-joint elastic coefficients as a combination of two elastic components with different properties, instead of considering them as having one single property over the entire range of joint angles. We first determined an optimal degree that divides the elastic coefficients of the elbow joint into two properties in healthy individuals and then divided the elastic coefficients into distal (from 10° to optimal segmentation degree) and proximal (from optimal segmentation degree to 110°) for comparison with the UPDRS rigidity score of PD patients.

## 2. Materials and Methods

### 2.1. Subjects

This study included 20 healthy elderly volunteers (15 men and 5 women, mean age of 71.2 ± 7.2 years) and 24 patients diagnosed with PD according to the British Brain Bank clinical criteria [3] (17 men and 7 women, mean age of 69.8 ± 7.6 years). Clinical details of the PD patients are shown in Table 1. PD patients were assessed using the UPDRS Part III, and rigidity was scored using the five-point scale [4] (0 = no rigidity, 1 = slight, 2 = mild to moderate, 3 = marked, and 4 = severe). The rigidity detected only during activation was not rated as score 1 or 0, because the patients were instructed to remain relaxed during the measurement and no movement was induced. All patients were on medication during the assessment of the UPDRS as well as the measurements, except for one patient (19) who was not taking any medication to treat the symptoms of PD. In our study, the UPDRS rigidity score was 1 or higher in all of the PD patients and 0 in all healthy subjects. This study was approved by the Institutional Review Board of Toneyama National Hospital and Osaka University Hospital, and written informed consent was obtained from all subjects. 

### 2.2. Protocols

**Figure 1:**
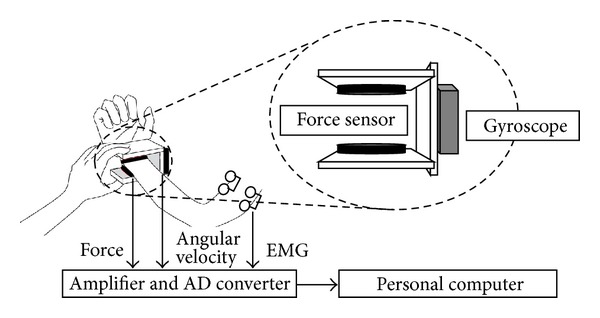
Schematic diagram of muscle tone measurement system. This system consists of small 3-axis force sensors, a gyro sensor, and surface electrodes. Two force sensors are placed to sandwich the wrist joint with soft pads to measure the force along the *z*-axis during flexion and extension movements of the elbow joint and calculate the torque at the elbow joint. The signals from the gyro sensor attached between the force sensors are used to calculate the angle of the elbow joint. The surface electrodes are applied to the muscle belly of the biceps and triceps brachii to record myoelectric activity.

Figure 1 shows an overview of a muscle tone measurement system. The measurement method used is as described in our previous published report [2]. The subjects were instructed to remain relaxed at rest in the sitting position, and an examiner held the elbow joint of the subject with one hand and the wrist joint of the subject with the other hand and gave passive flexion and extension of the elbow joint. The measurement was started from the maximum extension position. The subject was instructed to repeat the following movements for 60 sec: more than 3 sec rest, flexion over 2 sec, more than 3 sec rest at the maximum flexion position, extension over 2 sec, and more than 3 sec rest at the maximum extension position. Each trial included 4-5 cycles of flexion and extension, and 2 sets of the trial were performed for each upper limb. The data of the 7 out of 40 limbs of healthy subjects that potentially included apparent voluntary movements as determined by visual inspection of the surface electromyogram were excluded. Eventually, 260 measurements for each flexion and extension movement were obtained from healthy volunteers. In addition, 342 measurements for each of flexion and extension movements were obtained from PD patients.

### 2.3. Data Analysis

**Figure 2:**
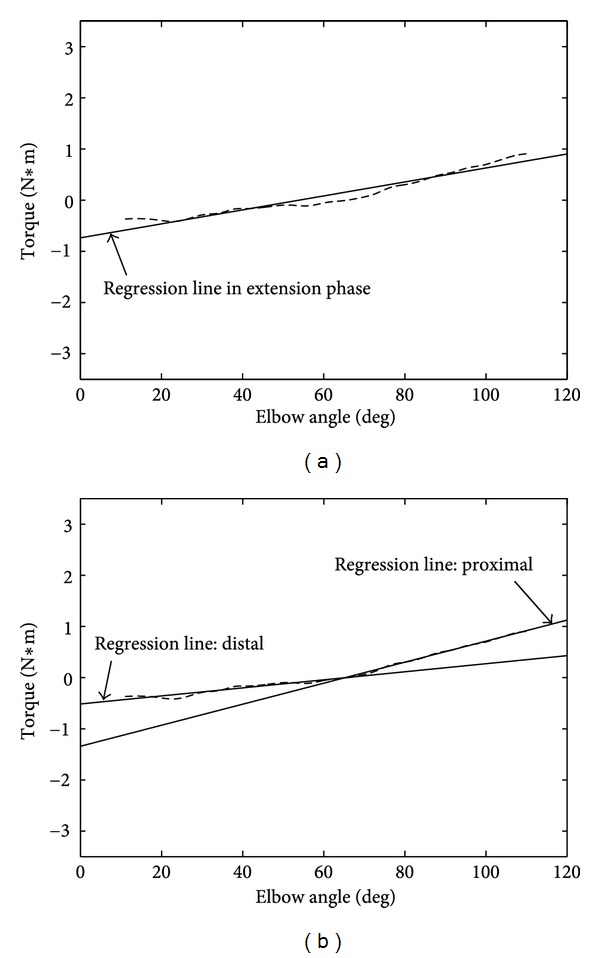
Methods of analysis of properties of elbow joint movements for each of the distal and proximal segments with the joint angle of “cutoff point” as their boundary. (a) One regression line was fitted to the data of the 10°–110° portion of the degree-torque characteristics curve obtained during extension of the elbow joint of a normal healthy subject. (b) Two regression lines were fitted to the data of the same subject. The extension of the elbow joint was divided into the proximal phase and distal phase at the angle of the elbow joint of  “cutoff point.”

(1) The 10°–110° portion of the degree-torque characteristics curve was extracted for each of the flexion and extension movements in healthy subjects.

(2) A likelihood ratio test was used to compare the fit of two regression lines: one of which (the null model) is one line fitting with no cutoff point and the other (the alternative model) is two lines fitting with a specified cutoff point. *P* values less than 0.05 were considered “significant,” which suggested that the two lines fitting with a specified cutoff point were better than one line fitting with no cutoff point (see Figures 2(a) and 2(b)).

(3) In “significant cases,” the degree of joint angle that divided the regression line was taken as a “cutoff point” and a 95% confidence interval (CI) for the cutoff point was obtained.

(4) The 10°–110° portion of the degree-torque characteristics curve was extracted for each of the flexion and extension movements in healthy volunteers and PD patients. For both flexion and extension, the regression line was divided by the cutoff point, and the “elastic coefficient in the distal phase” was calculated from the slope of the regression line from 10° to the cutoff point and the “elastic coefficient in the proximal phase” from the slope of the regression line from the cutoff point to 110°.

(5) The correlation between the UPDRS rigidity score and each value of the elastic coefficient for each of flexion and extension movements was evaluated. A pairwise comparison was made between score 0 group and score 1 group. The elastic coefficients in extension and flexion were normalized using the mass of the subject's body weight since these are dependent on the subject's muscle mass [5].

(6) We investigated whether a differentiation between healthy subjects and PD patients can be made with logistic discriminant analysis using two values of the elastic coefficient for each of the flexion and extension movements.

### 2.4. Statistical Analysis

Spearman's correlation coefficients with 95% CI were calculated to assess a degree of relationship between variables, and Wilcoxon rank sum test was used for pairwise comparisons. It could not be assumed that the raw data obtained were homoscedastic and normally distributed.

## 3. Results and Discussion

An analysis was performed for each of the 260 measurements in flexion and extension for healthy subjects. This analysis indicated that it is preferable for all the data to be approximated by two lines. The cutoff point was identified as 58.1° in flexion (95% CI: 55.3° to 60.9°) and 61.1° in extension (95% CI: 59.2° to 62.9°). The 95% CI for both cutoff points included 60°, and the cutoff points for both flexion and extension were determined as 60°.

**Figure 3:**
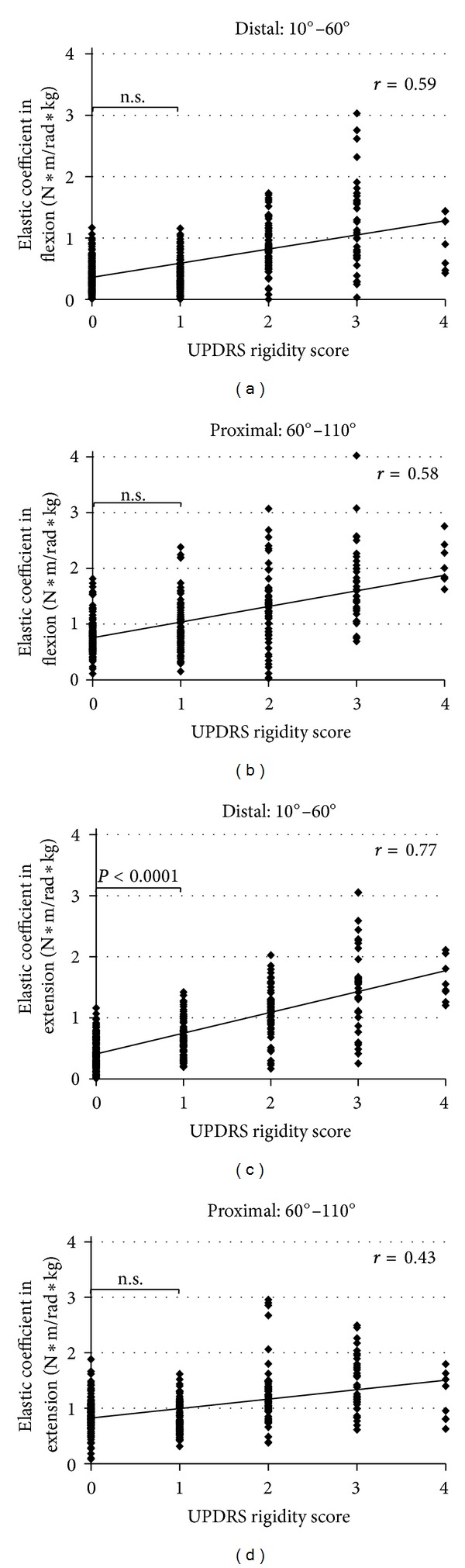
Results for elastic coefficients in elbow flexion and extension for each of distal and proximal phase. Comparison between the elastic coefficient in (a) the distal phase of flexion (10–60°), (b) the proximal phase of flexion (60–110°), (c) the distal phase of extension (10–60°), and (d) the proximal phase of extension (60–110°) and the UPDRS rigidity score. The elastic coefficient in the distal phase of extension showed the best correlation (*r* = 0.77) and demonstrated a significant difference between the UPDRS rigidity score 0 group and 1 group, whereas other three elastic coefficients did not show a significant difference between the groups.

Then, in healthy subjects and PD patients, the elastic coefficients in the distal phase (10°–60°) and proximal phase (60°–110°) were calculated with the cutoff point of 60° for 4 measurements each in flexion and extension. The correlation between these values and the UPDRS rigidity score was evaluated (see Figures 3(a), 3(b), 3(c), and 3(d)). The elastic coefficients in the distal phase of flexion (*r* = 0.59, 95% CI: 0.39–0.79), proximal phase of flexion (*r* = 0.58, 95% CI: 0.44–0.71), distal phase of extension (*r* = 0.77, 95% CI: 0.60–0.94), and proximal phase of extension (*r* = 0.43, 95% CI: 0.22–0.64) were all correlated with the UPDRS rigidity score. The best correlation for the elastic coefficient was found in the distal phase of extension. A significant difference between the UPDRS rigidity score 0 group and 1 group was observed in the elastic coefficient in distal phase of extension (*P* < 0.0001). No significant difference was observed in the proximal phase of extension (*P* = 0.1839), in the distal phase of flexion (*P* = 0.7495), or in the proximal phase of flexion (*P* = 0.1363).

A logistic discriminant analysis was performed, taking into account the calculated elastic coefficients, age, gender, and side (right or left), to investigate the feasibility of screening for PD. Among the 4 calculated elastic coefficients, those in the distal phase of extension, proximal phase of extension, and proximal phase of flexion were selected, and the analysis was performed with the addition of age. The results showed that healthy individuals and PD patients could be differentiated with 78.8% sensitivity, 83.3% specificity, and 81.5% correct classification rate, suggesting that it may be possible to perform screening for PD with high specificity.

As reported by Amis et al., moment arms vary substantially with joint angles in flexion and extension movements of the elbow joint in healthy individuals [6]. Moment arm properties have been shown to shift from approximately 40° for the biceps brachii, approximately 50° for the brachialis, and approximately 60° for the triceps brachii muscles. An et al. demonstrated through a simulation that the brachialis and biceps brachii may be responsible for the movements of the elbow joint in distal and proximal phases, respectively, with the joint angle of approximately 60° as the boundary between distal and proximal segments [7]. One can estimate that the elastic coefficients for flexion and extension of the elbow joint may change from the joint angle of approximately 60° based on these published reports. However, it is our precise measurement system that first demonstrated the validity of the method of analysis of properties of elbow joint movements for each of the proximal and distal segments with the joint angle of 60° as their boundary.

In the present analysis, we obtained 4 values of the elastic coefficients by calculating the coefficients divided into two blocks. Among them, the elastic coefficient in the distal phase of extension was best correlated with the UPDRS rigidity score. The combination that yielded the highest specificity in screening for PD was the elastic coefficients in the distal phase of extension, proximal phase of flexion, and proximal phase of extension. These results suggest that rigidity is clearly detected at the phase where the flexor and extensor muscle groups at the elbow joint are stretched from the middle and that rigidity is more pronounced in extension than in flexion. The Parkinsonian rigidity has been thought to be constant through a full range of joint angle [1], which may not be true if more detailed analyses were to be performed.

So far, rigidity has been rated as a score of 4 in the UPDRS rigidity score in individuals with limited joint mobility, and attention has been drawn to the distal phase of extension. This level of rigidity can be easily perceived by the human senses. At the level of score 2 or 3, difference in torque measurements for flexion and extension (difference of bias) is likely to be the major feature value, as described in our previous report. In our previous study, distinction between score 0 and 1 rigidity could be made by using continuous activity on the surface electromyogram at the maximum extension position of the muscle as a feature value; however, the results of the present study demonstrated that the feature value exists in the elastic coefficient in the distal phase of extension, which supports the findings of our previous study. 

The judgment of UPDRS rigidity score 1 (slight rigidity) seems to be based on the elastic component in the distal phase of extension. Further investigation for difference in muscle tone between rigidity and spasticity will be needed because it has been reported that Parkinsonian rigidity also has a spastic component which is described as “rigospasticity” [8].

## 4. Conclusions

Our study demonstrated that Parkinsonian rigidity shows variable properties depending on the elbow joint angle, and it is clearly detected at the distal phase of elbow extension. 

The scientific sensing technology has revealed that a feature to be noted is common to both the weakest and strongest rigidity. If such a simple and compact measurement system can be used at the bedside, new avenues will be opened for understanding the pathogenesis of the abnormality of muscle tone (including spasticity) and assessing treatment effects.

## Figures and Tables

**Table 1:** Patients' clinical details.

Patient	Age (yrs)	Gender	Disease duration (yrs)		UPDRS score		Medication*
				Part III	Rigidity (R)	Rigidity (L)	
1	79	M	4.5	30	2	1	C 2 mg; C/L 25/250 mg
2	85	M	1	21	1	1	Pra 0.375 mg
3	64	M	3	19	1	1	Pra 1.5 mg; C/L 20/200 mg
4	50	F	5.5	15	1	1	Per 0.9 mg; B/L 62.5/250 mg; T 1 mg
5	58	F	20	26	1	1	B/L 50/200 mg; A 200 mg; T 4.5 mg
6	76	F	8	39	2	2	C/L 20/200 mg
7	60	M	3.5	24	1	1	Pra 1.375 mg
8	77	F	4	29	1	1	Pra 1.5 mg; Per 0.15 mg
9	67	M	8	26	1	1	Pra 3 mg; Per 0.9 mg; B/L 75/300 mg; S 2.5 mg
10	70	F	4.5	43	1	1	Per 0.75 mg; C/L 20/200 mg
11	66	M	15	45	3	3	Per 0.75 mg; B/L 100/400 mg; E 400 mg; S 5 mg; T 4 mg
12	73	F	12.5	32	1	1	Pra 1.5 mg; Per 1.05 mg; B/L 75/300 mg; S 5 mg
13	58	M	9	28	2	3	Pra 1.5 mg; Per 0.75 mg; C/L 15/150
14	67	M	8.5	39	2	1	Per 0.75 mg; B/L 150/600; S 2.5 mg; T 2 mg
15	67	M	5	53	2	2	C 3 mg; C/L 15/150 mg
16	78	M	5.5	35	2	2	Pra 3 mg; B/L 75/300
17	47	M	9	24	1	2	Pra 2 mg; C 2 mg; B/L 50/200; A 300 mg
18	67	M	5.5	39	1	2	Pra 1.5 mg; Per 0.6 mg; C/L 30/300
19	67	F	3	45	2	3	No medication
20	69	M	4	49	4	3	B/L 25/100
21	72	M	5	49	2	3	Pra 1.5 mg; B/L 125/500
22	60	F	8	18	2	3	Pra 2 mg; C 1 mg; C/L 15/150
23	73	M	6	51	4	3	Per 0.15 mg; B/L 62.5/250; A 125 mg; T 2 mg
24	65	F	2.5	29	3	2	Pra 0.75 mg; C/L 10/100; S 2.5 mg; D 200 mg

*C: cabergoline; C/L: carbidopa/levodopa; Pra: pramipexole; Per: pergolide; B/L: benserazide/levodopa; T: trihexyphenidyl; A: amantadin; S: selegiline; E: entacapone; D: droxidopa.
